# Registered multi-device/staining histology image dataset for domain-agnostic machine learning models

**DOI:** 10.1038/s41597-024-03122-5

**Published:** 2024-04-03

**Authors:** Mieko Ochi, Daisuke Komura, Takumi Onoyama, Koki Shinbo, Haruya Endo, Hiroto Odaka, Miwako Kakiuchi, Hiroto Katoh, Tetsuo Ushiku, Shumpei Ishikawa

**Affiliations:** 1 Department of Preventive Medicine, Graduate School of Medicine, The University of Tokyo, 7-3-1 Hongo, Bunkyo-ku, Tokyo, 113-0033 Japan; 2 Division of Gastroenterology and Nephrology, Department of Multidisciplinary Internal Medicine, School of Medicine, Faculty of Medicine, Tottori University, 36-1 Nishicho, Yonago, Tottori, 683-8504 Japan; 3 Department of Pathology, Graduate School of Medicine, The University of Tokyo, 7-3-1 Hongo, Bunkyo-ku, Tokyo, 113-0033 Japan; 4 Division of Pathology, National Cancer Center Exploratory Oncology Research & Clinical Trial Center, 6-5-1 Kashiwanoha, Kashiwa, Chiba, 277-8577 Japan

**Keywords:** Pathology, Medical imaging

## Abstract

Variations in color and texture of histopathology images are caused by differences in staining conditions and imaging devices between hospitals. These biases decrease the robustness of machine learning models exposed to out-of-domain data. To address this issue, we introduce a comprehensive histopathology image dataset named PathoLogy Images of Scanners and Mobile phones (PLISM). The dataset consisted of 46 human tissue types stained using 13 hematoxylin and eosin conditions and captured using 13 imaging devices. Precisely aligned image patches from different domains allowed for an accurate evaluation of color and texture properties in each domain. Variation in PLISM was assessed and found to be significantly diverse across various domains, particularly between whole-slide images and smartphones. Furthermore, we assessed the improvement in domain shift using a convolutional neural network pre-trained on PLISM. PLISM is a valuable resource that facilitates the precise evaluation of domain shifts in digital pathology and makes significant contributions towards the development of robust machine learning models that can effectively address challenges of domain shift in histological image analysis.

## Background & Summary

Since the 1990s, whole-slide scanners have facilitated the capture of high-resolution digital images of complete specimens, similar to microscopic images. This has led to the development of digital pathology, which employs computers to analyze whole-slide images (WSIs). Along with rapid advancements in deep learning, researchers are developing artificial intelligence (AI) to help minimize the workload of pathologists, aid in predicting patient prognosis, and provide decision support for treatment plans based on WSIs^1^.

However, unwanted color and texture heterogeneity^1–3^ is present in digital histology images. This heterogeneity is the primary cause of a domain shift in pathological images, thereby restricting the clinical application of deep learning algorithms by decreasing their generalizability^4^. Heterogeneity results from the inconsistencies in the procedures before obtaining WSIs, such as tissue preparation, staining, and scanning^5,6^. For instance, inconsistencies in the formulations of hematoxylin and eosin (H&E), exposure to light, and varying storage conditions lead to color inconsistencies^7^. Additionally, different scanners have unique imaging properties, resulting in color and texture variations^7^. Histology images captured through microscopes using smartphones also add to this variability. Smartphones are widely used to capture histological images as they enable the pathologists to easily consult with colleagues, seek consensus, and share images of interest^8,9^. This trend is particularly pronounced in developing countries, where resources might be limited^10^. Moreover, mobile tools such as content-based image retrieval, which provide image similarity search capabilities, have emerged to address the growing demand for assistance in pathological diagnosis using mobile phones^11,12^. Nevertheless, images taken by smartphones significantly differ in quality from those produced by WSI scanners. Furthermore, the wide variety of smartphone devices contributes to the variability in image quality, thereby exacerbating the problem of color and texture heterogeneity.

Color augmentation^1,13,14^ is a common technique used to enhance the robustness and generalizability of deep-learning models against color variation. Although this augmentation involves applying random changes in hue, saturation, and brightness to the input image, the degree of perturbation is a hyperparameter that is difficult to optimize. Small perturbations are ineffective at increasing the robustness. Contrarily, large perturbations may lead to an unnatural color distribution, causing a drop in the performance of the trained model^14^. While deep learning-based style transfer methods have been utilized to improve robustness against both color and texture variations, histological images in diverse domains are required to train the model^15^. Therefore, histopathological datasets encompassing various domains could be beneficial for researchers to optimize the color range of augmentation or develop robust style-transfer models.

Recent studies have provided histopathological image datasets for various domains. For instance, the MIDOG++^16^ dataset includes 503 cases of H&E-stained WSIs from seven types of cancer. The images were obtained using five distinct WSI scanners to detect mitotic cells effectively. Kuritcyn and colleagues^17^ created an image dataset consisting of 161 cases of colorectal cancer images captured using six different scanners. They found that, model performance decreased owing to domain shifts. The CAMELYON dataset^18^ was developed to construct a model aimed at detecting tumor metastases in sentinel lymph nodes, although it was not primarily designed to address domain shifts. The dataset comprises lymph node specimens, scanned using three distinct scanners from multiple medical institutions. However, these studies have several limitations: (1) they target only one organ, except for MIDOG++, which results in a lack of tissue diversity in the images obtained; (2) these studies are limited to only WSI scanners; (3) they do not focus on the differences in staining conditions, which results in a lack of diversity in H&E staining; and (4) the same tissues are not captured across domains, thus limiting the ability to evaluate color and texture differences between different domains. Kuritcyn’s dataset is an exception; however, it is not publicly available. The features of each dataset are summarized in Table 1.

**Table 1:** Comparison of the existing datasets for domain adaptation with PathoLogy Images of Scanners and Mobile phones (PLISM).

	MIDOG++	Kuritcyn^17^	CAMELYON	PLISM (ours)
images from WSIs	✓	✓	✓	✓
smartphone images	×	×	×	✓
multi-tissue	✓	×	×	✓
multi-stain	×	×	×	✓
same tissue	×	✓	×	✓
availability	✓	×	✓	✓

**Fig. 1:**
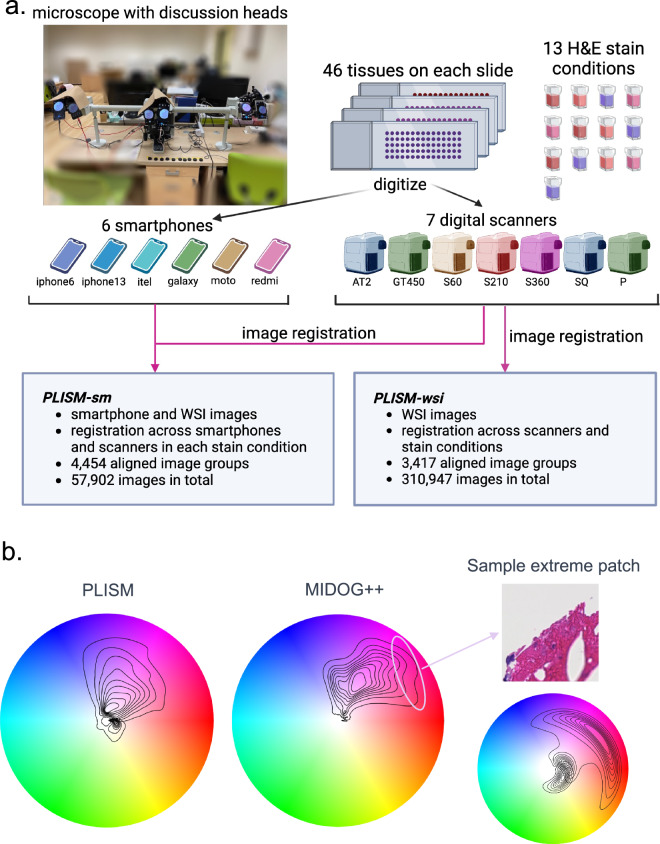
(**a**) Pathology Images of Scanners and Mobile phones (PLISM) Workflow, and b) Comparison of HSV color wheels between PLISM (left) and MIDOG++ (right) images. (**a**)Tissue microarray slides containing 46 tissues stained under thirteen different staining conditions were scanned with six smartphones and seven slide scanners (objective lens at 40×). To ensure that the same field of view of the tissue was captured by all smartphones, each smartphone was attached to an eyepiece of the discussion microscope. Image registration was performed across all imaging modalities using smartphone images as the query (*PLISM-sm* subset) and throughout only WSIs’ images (*PLISM-wsi* subset). (**b**) In the left and center figures, kernel density estimation plots represent data distribution of 512 × 512 px RGB images resized to 50 × 50 px (n = 10,000), randomly extracted from each dataset. The right figure shows a plot of hue and saturation of pixels from regions with an extremely strong red hue.

To address this issue, we developed a dataset named Pathology Images of Scanners and Mobile phones (PLISM)^19^ (Fig. 1a). The dataset contains histopathological images from various domains, including different tissue types, staining conditions, and imaging devices. It covers a wide range of colors similar to that of MIDOG++, comprising images of specimens obtained from multiple laboratories, except for the spectrum with an extremely strong red hue. Based on the observation of a high prevalence of artifact images within the strongly red-hued section of the MIDOG dataset, we believe that the data distribution of the PLISM dataset aligns with existing external datasets (Fig. 1b). The strength of the PLISM dataset lies in its unique design, in which images encompass both WSIs and smartphone images that capture the same tissue or serial sections of tissue microarray (TMA) stained under different H&E staining conditions. Each TMA slide contained 46 different tissues from the human body, providing a diverse tissue collection. We aligned these images properly from different domains at the patch level, which allowed for the statistical analysis of the imaging modality and staining types. This dataset can help evaluate the robustness of an AI model in various domains, providing valuable insights into the impact of diverse imaging modalities and staining on the algorithms. To the best of our knowledge, the PLISM dataset is the first of its kind to encompass a diverse collection of H&E-stained images captured using multiple imaging modalities, such as smartphones, and obtained using various hematoxylin solvents.

## Methods

### Data collection

**Fig. 2:**
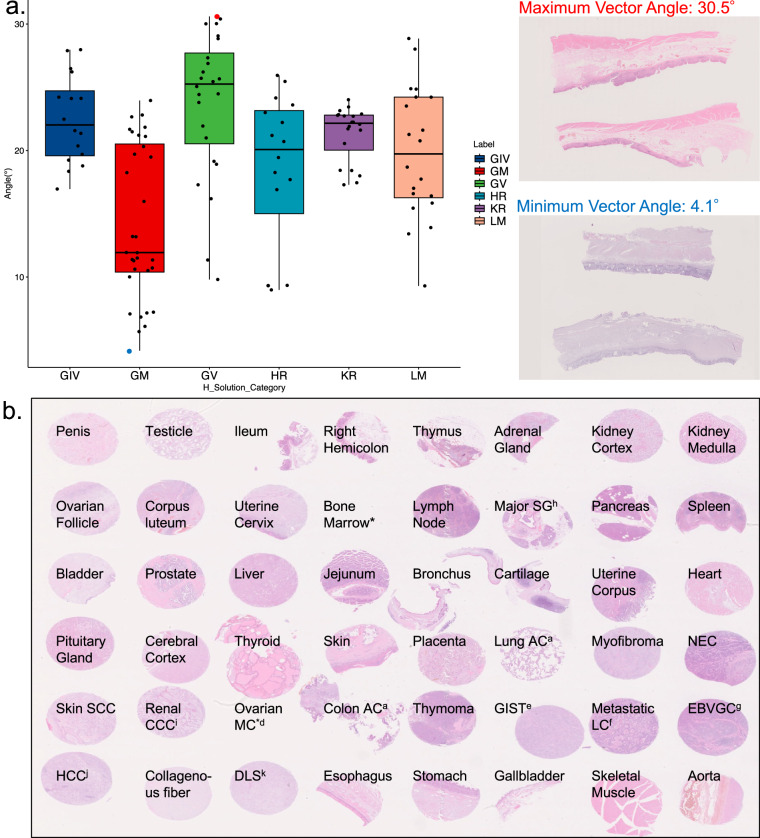
(**a**) Angles formed by hematoxylin and eosin (H&E) component vectors for each of the 64 staining conditions, and (**b**) Tissue types included in tissue microarray (TMA) specimens. (**a**) Angles are categorized by the type of hematoxylin solvent group. Red and blue points in the boxplot indicate the maximum and minimum angles, respectively, with corresponding images shown on the right. (**b**) a. Adenocarcinoma. b. Neuroendocrine carcinoma. (**c**) Squamous cell carcinoma. (**d**) Mucinous carcinoma. (**e**) Gastrointestinal stromal tumor. (**f**) Liver cancer. (**g**) Epstein-Barr virus-positive gastric cancer. (**h**) Salivary gland. (**i**) Clear cell carcinoma. (**j**) Hepatocellular Carcinoma. (**k**) Dedifferentiated liposarcoma. *This tissue was lost due to detachment from the slide during slicing.

All histopathological specimens used in creating the PLISM dataset were sourced from patients who were diagnosed and underwent surgery at the University of Tokyo Hospital between 1955 and 2018. This study was approved by the Institutional Review Board of the University of Tokyo (approval number: 2381). Each TMA slide consisted of 46 different tissues extracted from formalin-fixed, paraffin-embedded human tissues, as shown in Fig. 2.

From a pool of 64 H&E staining conditions, we selected two staining conditions for each H solution category along with the MY staining condition routinely used in our laboratory. The selection criteria were based on the color similarity between H and E, selecting an H solution with the highest color similarity to E and another with the lowest color similarity to E. This approach ensured a color diversity. Specifically, the stain deconvolution method was used to deconvolve the RGB color of the histology image into the H and E color vectors. Subsequently, for each H solution category, the H solution with the minimum and maximum angle to the E color vector was selected. A greater contrast was achieved when the angle between the two vectors was larger, resulting in a vivid color appearance with a clear distinction between the H and E components (e.g. GIVH, GMH, GVH, HRH, KRH and LMH in Table 2 Abbrev.). Conversely, a smaller angle produced a darker tone (e.g. GIV, GV, GM, GV, HR, KR and LM in Table 2 Abbrev.) (Fig. 2a). Therefore, these criteria contributed to the diversity of H&E staining in the dataset. Out of these staining conditions, H was exposed for approximately 24 hours in two conditions, which is considered impractical in clinical settings. These staining conditions were selected to generate an extreme reference H color distribution. A total of 13 slides were stained using the 13 selected H&E staining conditions (Table 2).

**Table 2:** Hematoxylin and eosin (H&E) staining conditions used in PLISM.

Type of Hematoxylin (product number)	Exposure time of Hematoxylin (min)	Exposure time of Eosin (min)	Number of Dehydration	Abbrev.	Solution Category
Gill IV (8647)	0.5	15	1	GIVH	GIV
Gill IV (8647)	Overnight*	15	4	GIV	
GM (30081)	2	15	1	GMH	GM
New Type G (30161)	5	15	4	GM	
Gill V (20032)	5	15	1	GVH	GV
Gill V (20032)	60	15	4	GV	
Mayer (30002)	3	3	1	MY	MY
Harris (20022)	2	15	1	HRH	HR
Harris (20022)	Overnight*	15	1	HR	
Carrazi (30131)	5	15	1	KRH	KR
Carrazi (30131)	60	15	4	KR	
Lillie-Mayer (30072)	2	15	1	LMH	LM
Lillie-Mayer (30072)	2	15	5	LM	

*About 24 hours.

### Slide digitalization, image registration, and image tiling

The workflow, from slide digitization to image registration are presented in Fig. 1. Once the slides were stained with H&E, they underwent digitization using seven slide scanners and six smartphones to capture scan variability across the devices used, in addition to the variability in the H&E staining procedure. H solvents used were produced by either Sakura Finetek Japan Co., Ltd. or Muto Pure Chemicals Co., Ltd. (Tokyo, Japan). The scanners used in the PLISM are listed in Table 3. All slides were scanned at the maximum resolution of each scanner used, which ranged from 0.22 to 0.26 µm/pixel.

**Table 3:** Whole-slide image (WSI) scanners used in the dataset.

Vendor	Model	Abbrev.	Year Released	MPP (µm/pixel)	File Format
Hamamatsu	NanoZoomer-S360 C13220-01 scanner	S360	2017	0.229	ndpi
Hamamatsu	NanoZoomer-S210 C13239-01 scanner	S210	2015	0.220	ndpi
Hamamatsu	NanoZoomer-SQ C13140-D03	SQ	2014	0.221	ndpi
Hamamatsu	NanoZoomer-S60 C13210-01	S60	2016	0.220	ndpi
Leica	Aperio AT2	AT2	2013	0.253	svs
Leica	Aperio GT450	GT450	2019	0.262	svs
Phillips	Ultrafast Scanner	P	2018*	0.25	isyntax

*Release year in Japan.

We used discussion heads attached to an Olympus BX-53 microscope (EVIDENT Co. Ltd., Tokyo, Japan) to capture the same microscopic images using various smartphones (Fig. 1a). Each discussion head was attached to a different smartphone. The smartphones used in PLISM are listed in Table 4. All images were captured at 400 × magnification of the microscope. The Open Camera^20^ tool was used for the Android smartphones, whereas the Camera + 2^21^ tool was employed for the iPhones. Images were captured by manually pressing a Bluetooth switch (ELECOM, P-SRBBK 4953103305977). This setup allowed images of the same tissue structure to be captured simultaneously without the influence of motion blurring from the capturing process. The imaging settings for each tool and camera specifications for each smartphone are presented in Table 5.

**Table 4:** Specification for smartphone cameras.

Vendor	Model	Number of Pixels (Megapixels)	Resolution (pixels)	F-number (f)
Samsung	Galaxy S20 5 G SC-51A	12	1440 × 3200	1.8
Motorola	moto g8	16	720 × 1560	1.7
Xiaomi	Redmi Note9 Pro	64	1080 × 2400	1.9
Apple	iPhone 6	8	750 × 1334	2.2
Apple	iPhone 13 mini	12	1080 × 2340	2.4
Transsion	iTel P33	8	720 × 1440	1.8

When the smartphone used had multiple lenses, the information about the lens actually used for capturing the image is provided.

**Table 5:** Imaging setting details for applications.

Tool	Camera + 2	Open Camera
Focus	Manual	Manual
Flash	No	No
Exposure	Automatic Exposure	Fixed
White Balance	Auto	Auto
File Format	JPEG (for iPhone 6) TIF (for iPhone 13 mini)	PNG

All slides were initially stored in the original vendor format. Subsequently, we used VALIS^22^, an open-source image registration package, to perform both rigid and non-rigid registration among WSIs, designating the slide scanned by Hamamatsu Nanozoomer S60 with Mayer stain as the reference slide and setting ‘align_to_reference’ parameter as True.

We created two PLISM subsets from the images:

1. *PLISM-wsi* contains only WSI images. Registration was performed across all scanners and staining conditions. There were 3,417 aligned image groups, with a total of 310,947 (3,417 groups × 91 WSIs) image patches.
2. *PLISM-sm* includes both smartphone and WSI images. Registration was performed on all scanners and smartphones under each staining condition. There were a total of 4,454 aligned image groups containing 57,902 (4,454 groups × 13 devices) images.

**Fig. 3:**
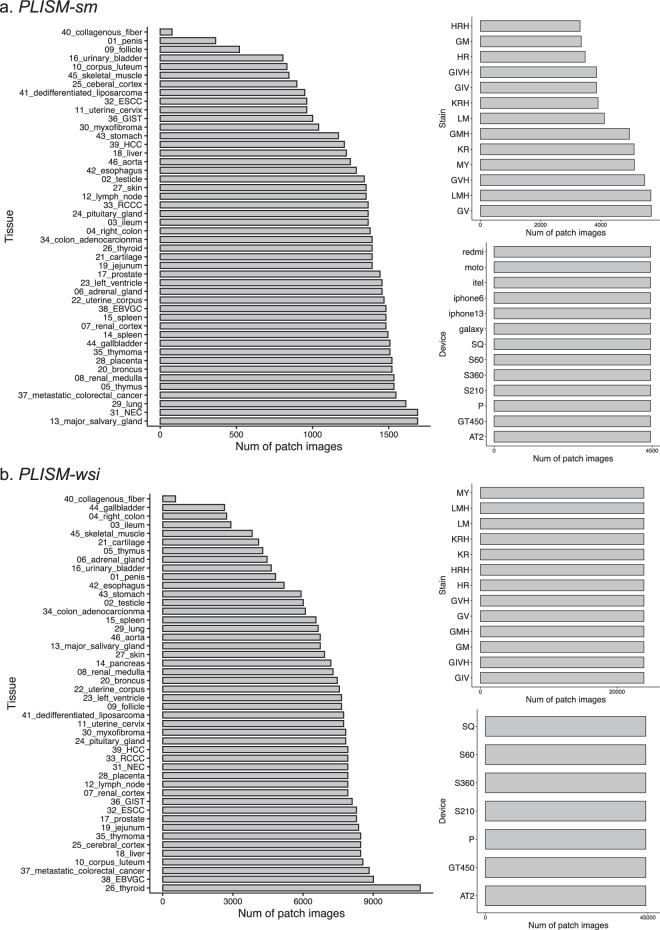
The number of images per tissue, staining condition, and imaging device in (**a**) *PLISM-sm* and (**b**) *PLISM-wsi*.

**Fig. 4:**
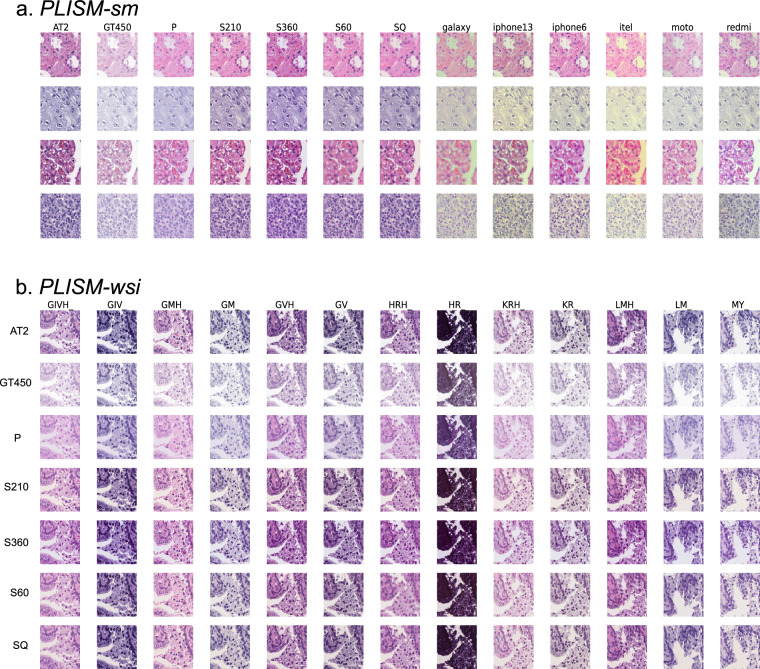
Example images of (**a**) *PLISM-sm* and (**b**) *PLISM-wsi*. Since different staining cannot be applied to stain the same sections, we stained the serial sections, resulting in a slightly different tissue appearance between different staining conditions while preserving the tissue components.

For the *PLISM-sm* subset, smartphone images were used as queries, and employed OpenCV’s AKAZE^23^ key point matching algorithm to extract the corresponding tissue regions from each WSI with a matching stain type. In *PLISM*-*sm*, the size of the reference WSI images varied for each smartphone image. Rigid registration was then performed twice to align the WSI and the smartphone images using ‘cv2.findHomography’ and ‘cv2. warp Perspective’ with RANSAC^24^ filtering. After image alignment, each image was center-cropped to a size of 512 × 512 pixels because we observed large registration errors around the edges of the images. For the *PLISM-wsi* subset, we tiled all 91 WSIs to 1024 × 1024 pixels without overlap, regardless of staining. Subsequently, we again performed rigid registration on the AT2, GT450, and P scanner images using OpenCV’s AKAZE^23^ key-point matching algorithm to align them with the S60 scanner images, because of their relatively large visible misalignment. Each image was center-cropped to a size of 512 × 512 pixels. Figure 3 shows the number of images classified by tissue type, staining type, and imaging device for both subsets. Example images of both subsets are presented in Fig. 4.

### Data validation and quality control

All glass slides included in the PLISM dataset were manually stained and scanned by M. O. and experienced technical staff. The staining quality was visually confirmed by a board-certified pathologist (M. O.), and all images were manually inspected after tiling the WSIs. The quality of the tiled images was assessed by technical staff (H. E. and K. S.), and tile-cropped images with missing parts or significant focus problems were excluded from the analysis.

The registration quality was also evaluated. The Target Registration Error (TRE), the median distance between the registration target features in the image, and the corresponding matched features^22^ in the reference image was 43 μm for registration between serial sections. This value is within the normal range for serial sections where the target points can be shifted between sections. As Gatenbee *et al*. demonstrated in the original VALIS study^22^, the TRE between serial WSIs after both rigid and non-rigid registration is approximately between 20 μm and 100 μm. Furthermore, to evaluate the quality of registration between WSIs and smartphone images, we manually refined the position of each landmark such that it was positioned on the same cell or prominent tissue landmarks in the corresponding WSI-smartphone-group images. In total, we created and manually checked 325 registration points on 65 patch images, which resulted in a 1.0 μm TRE score. All groupwise images were manually checked by H.E., K.S., and O.M., and misaligned image groups were removed.

### Evaluation methods

To test whether the PLISM can improve the robustness of convolutional neural networks (CNN) to out-of-domain datasets and exceed conventional color augmentation, we pretrained two ResNet18s model using SimCLR^25^, a self-supervised learning method. The PLISM-full model was pretrained on *PLISM-sm* dataset, while the PLISM-WSIonly model was pretrained on *PLISM-wsi* dataset. The two models used on the PLISM were pre-trained with 224 × 224 pixel images from each data subset for 1000 epochs using the same augmentation method as Ciga *et al*.^26^. The pretrained models were trained with a batch size of 256 and a learning rate of 0.3 × (batch size)/256. For a comparative evaluation, we also assessed CNN models trained on two different datasets: one featuring over one million general images from ImageNet with and without HED-light color augmentation method that demonstrated the best performance across various histology datasets^1^ and the other comprising of 57 histology datasets from the study conducted by Ciga *et al*.^26^. The latter included images from various organs, captured at resolutions ranging from 10 × to 100 × , and predominantly stained with H&E.

We evaluated the pretrained model on two multiclass classification datasets for colorectal adenocarcinoma: Kather19^27^ and CRC-TP^28^, and a binary classification dataset for the presence or absence of breast cancer metastasis in sentinel lymph nodes: Camelyon17^18^. For the colorectal adenocarcinoma datasets, we modified the datasets to focus exclusively on the six common classes in both the Kather19 and CRC-TP datasets: debris (DEB), lymphocytes (LYM), muscle (MUS), normal glands (NORM), simple stroma (STR), and tumor epithelium (TUM).

In the training for the downstream task, only the linear classification layer was trained, whereas the remaining layers were frozen to rigorously evaluate the performance of the pretrained model. We then evaluated the classified images against the ground truth labels and computed the mean F1 score across all test patch images of each tissue type. For the colorectal adenocarcinoma datasets, Kather19 was used for training and CRC-TP was used for testing. Next, CRC-TP was used for training and Kather19 was used for testing. The Camelyon17 dataset included cases from five institutions and we tested 10 combinations of institutions for the training and testing datasets. We trained the linear layer 10 times with random initial weights for each training and testing combination for the colorectal adenocarcinoma and breast cancer metastasis datasets.

### Statistical analysis

Data were analyzed using Python version 3.8.5 and R version 4.3.1. Statistical significance was assessed using Welch’s t-test with Bonferroni correction and two-way analysis of variance (ANOVA) test using “SciPy” library (“Quality Control for Differences in Color Variation Across Staining Conditions and Imaging Devices in Identical Tissue Images”), Kruskal–Wallis test with “ggpubr” library (Fig. 7). All statistical tests were two-sided and p value < 0.05 was considered statistically significant.

## Data Records

### Image data & lists of data

The entire PLISM dataset is publicly available on figshare plus under the CC-BY 4.0 license^19^. PLISM-wsi and PLISM-sm were deposited separately. The folder structure for each subset is as follows.

1. *PLISM-wsi*, consists of image groups for all staining conditions between the WSIs for each tile image. Image groups from the same field of view in the WSI images shared common coordinates in their filenames.

├(stain_name)_(device_name)/

└(stain_name)_(device_name)_(top_left_x)_(top_left_y).png

List of images selected through quality control by visual assessment. This is a CSV file with the following columns providing information regarding the images:

- **Tissue Type:** The tissue types out of the 46 types of human tissue.
- **Stain Type:** The staining condition out of the 13 types.
- **Device Type:** The device types out of the 13 device types.
- **Coordinate:** The xy coordinates of the upper-left corner of each WSI image (e.g., 1000 _500)*.
- **Image Path:** The relative path to each image file.

2. *PLISM-sm*, where smartphone images are used as queries to create image groups for each staining condition corresponding to each tile image. Image groups from the same field of view shared common coordinates in their file names, which corresponded to the WSI coordinates captured using the AT2 device under the respective staining conditions.

├(stain_name)/

└(device_name)/

└(top_left_x)_(top_left_y)_(right_lower_x)_(right_lower_y).png

List of images selected through quality control by visual assessment. This is a CSV file with the following columns providing information regarding the images:

- **Tissue Type:** The tissue types out of the 46 types of human tissue.
- **Stain Type:** The staining condition out of the 13 types.
- **Device Type:** The device types out of the 13 device types.
- **Coordinate:** The xy coordinates of the upper left and bottom right corners of each WSI image (e.g., 10000_5000 104000_9000)*.
- **Image Path:** The relative path to each image file.

All images were saved in PNG format. Original 91 WSIs are also publicly available^19^. The asterisks (*) indicate the coordinates of each image before they were center-cropped.

## Technical Validation

### Quality control for differences in color variation across staining conditions and imaging devices in identical tissue images

**Fig. 5:**
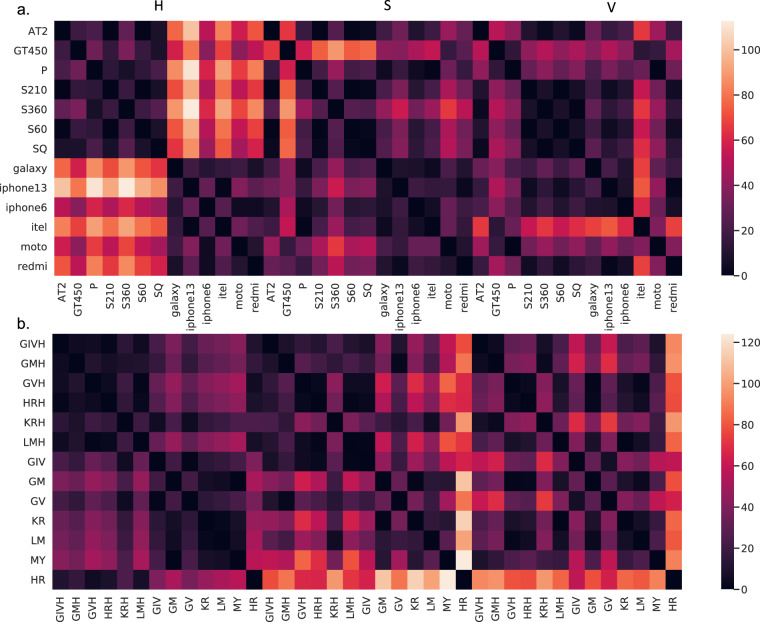
T-values for mean Hue, Saturation, Value (HSV) values by (**a**) Device type, and (**b**) Staining condition. The color scale indicates the absolute t-value in unpaired t-tests for each device and staining condition regarding the mean HSV values in the *PLISM-sm* subset.

For quality control, and to demonstrate the diversity of color and texture in the H&E-stained images of PLISM across staining conditions and imaging devices, we utilized the *PLISM-sm* subset, which includes all device types and captures the same tissue image for each group. We statistically tested the differences in color distribution for each of the Hue, Saturation, Value (HSV) components between different devices and staining conditions. Among the different devices, 216 (92.3%) of the 234 combinations were significantly different after the Bonferroni correction. Similarly, among the different staining conditions, 215 (91.8%) of 234 combinations were significantly different. These results suggest that almost all devices and staining types exhibited different color characteristics. The differences in HSV values according to the device and staining type are presented in Fig. 5. There was a greater difference in hue between smartphones and WSIs than between the WSI combinations. There was also a slight difference in the saturation between the WSIs and smartphones. When staining was examined, there was a similar trend of differences in hue and saturation between smartphones and WSIs; however, saturation showed a greater difference. As expected, HR staining with overnight H exposure had a strong H component, resulting in substantially different saturation and values compared to other staining types. However, GIV staining with a different overnight H exposure had a smaller difference in color.

To determine whether the differences in the HSV components were attributable to staining or device type, we compared the sum of squares using ANOVA for staining and device type. The analysis indicated that device type contributed more significantly to Hue (Device: Stain, 947.5 vs. 332.7, p < 0.000001), whereas staining contributed more to saturation (Stain: Device, 360.3 vs. 165.7, p < 0.000001) and value (Stain: Device, 318.9 vs. 120.7, p < 0.000001). This finding suggests that despite staining with various H solvents, the influence of device type on defining image coloration was greater than that of staining in our dataset.

**Fig. 6:**
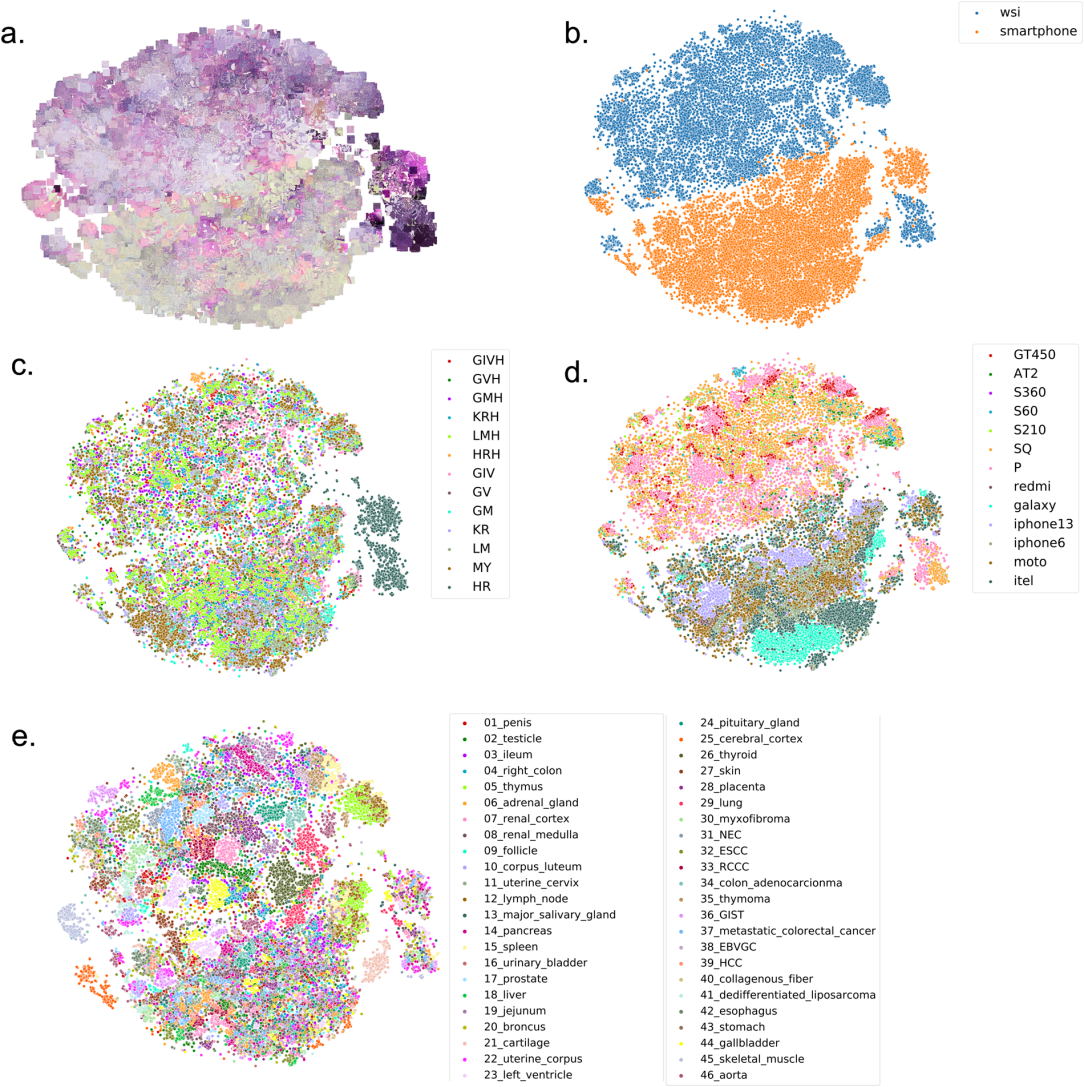
t-distributed Stochastic Neighbor Embedding (t-SNE) plot for the *PLISM-sm* subset. Each image was encoded using deep texture representations (DTR) method^11^ with the VGG16 model pre-trained on ImageNet. For the *PLISM-sm* subset, we calculated the 1024-dimensional DTRs of each image, performed dimensionality reduction using t-SNE from python sklearn library with learning_rate = ‘auto’, and plotted the results in 2D. The figure shows the t-SNE plots for (**a**) WSI and smartphones, (**b**) original images, (**c**) staining types, (**d**) device types, and (**e**) tissue types.

Subsequently, we assessed the color and texture differences in the feature space produced by the CNN model (Fig. 6). Interestingly, the images captured by the smartphones and the WSIs were clearly divided into two distinct clusters (Fig. 6a,b). While HR staining with overnight hematoxylin exposure formed a separate cluster, no clear clusters were formed for the other staining types or devices (Fig. 6c,d). However, a closer examination of the WSI cluster in Fig. 6e reveals that the images are loosely grouped by tissue type. These results suggest that the broad categories of devices, such as WSIs and smartphones, as well as tissue types, have distinct color tones and textures in the images.

### Improving domain shift using plism pre-trained convolutional neural network

**Fig. 7:**
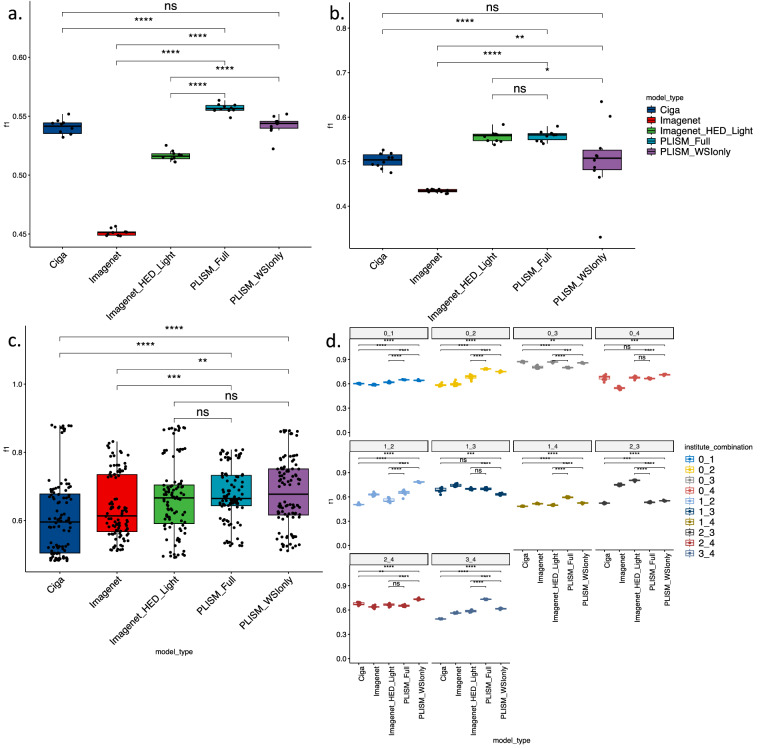
Out of distribution performance of convolutional neural network (CNN) models trained on various image datasets. Macro F1 scores, where (**a**) Kather19 was used as the training dataset and CRC-TP as the test dataset and (**b**) CRC-TP was used as the training dataset and Kather19 was used as the test dataset. Training and inference were performed 10 times for both (**a** and **b**). For the Camelyon17 dataset, combinations of training and test datasets were created for 10 facility combinations, and (**c**) the macro F1 scores for all combinations. (**d**) Macro F1 scores for each combination of training and test datasets. Training and inference were performed 10 times for each combination. In the box plot, the lower and upper hinges correspond to the 25th and 75th percentiles, respectively, and the upper whisker extends from the hinge to the largest value no further than 1.5 × interquartile range (IQR) from the hinge. The lower whisker extends from the hinge to the smallest value at most 1.5 × IQR of the hinge. ns: p > 0.05, *p < = 0.05, **p < = 0.01, ***p < = 0.001, ****p < = 0.0001.

We performed two sets of experiments: first, we used Kather19 for training and CRC-TP for testing (Fig. 7a) and second, we reversed the roles, employing CRC-TP for training and Kather19 for testing (Fig. 7b).

As shown in Fig. 7a, the model pre-trained on the *PLISM-sm subset* (PLISM-full) significantly outperformed both Imagenet with and without HED-light color augmentation method and Ciga’s models in terms of macro F1 scores (p ≤ 0.0001). In contrast, no significant differences were observed when comparing the model pretrained on the *PLISM-WSI* subset (PLISM-WSI only) with model by Ciga *et al*. In Fig. 7b, for the performance of ImageNet without the HED-Light augmentation method and Ciga’s model, we observed results similar to those observed in Fig. 7a when compared to that of the PLISM models. However, the ImageNet without HED-Light augmentation method and the PLISM-full model demonstrated comparable F1 scores.

We also tested the model using Camelyon17. This dataset included cases from five institutions, and we tested 10 combinations of institutions for the training and test datasets. As shown in Fig. 7c, both PLISM_full and PLISM_WSI had significantly higher F1 scores than ImageNet without HED-light augmentation method and Ciga models (p < = 0.001), and presented comparable scores compared to ImageNet with HED-Light augmentation method. For each combination shown in Fig. 7d, the model pretrained on PLISM significantly outperformed the other models in terms of F1 scores in seven of the 10 combinations. These results suggest that PLISM effectively simulates pathological images across various domains, and pretrained models using PLISM data have the potential to outperform conventional color augmentation methods when used independently.

## Data Availability

All codes used in the image registration between WSI and smartphone images described in the manuscript were written in Python 3 and are available through our GitHub repository (https://github.com/p024eb/PLISM-registration). We have provided all the necessary libraries and python scripts that allow the tracing of our results.
